# Special Issue: 100 years of scientific excavations at UNESCO World Heritage Site Monte San Giorgio and global research on Triassic marine Lagerstätten

**DOI:** 10.1186/s13358-024-00328-3

**Published:** 2024-10-01

**Authors:** Christian Klug, Torsten M. Scheyer, Nicole Klein, Jun Liu, Daniele Albisetti, Heinz Furrer, Rudolf Stockar

**Affiliations:** 1 Universität Zürich, Paläontologisches Institut, Karl-Schmid-Strasse 4, 8006 Zurich, Switzerland; 2 Abteilung Paläontologie, Institut für Geowissenschaften, Universität Bonn, Nußallee 8, 53115 Bonn, Germany; 3 School of Resources and Environmental Engineering, Hefei University of Technology, Hefei, 230009 China; 4 Chengdu Center, China Geological Survey, Chengdu, 610081 China; 5 State Key Laboratory of Palaeobiology and Stratigraphy, Nanjing Institute of Geology and Palaeontology, CAS, Nanjing, 210008 China; 6 Museo dei Fossili del Monte San Giorgio, Via Bernardo Peyer 9, 6866 Meride, Switzerland; 7 Repubblica e Cantone Ticino, Dipartimento del Territorio, Museo Cantonale di Storia Naturale, Viale Carlo Cattaneo 4, 6900 Lugano, Switzerland

**Keywords:** Marine reptiles, Fishes, Exceptional preservation, Konservat-Lagerstätten, Taphonomy, Triassic, Permian–Triassic mass extinction

## Abstract

Only a few Swiss fossil localities are known globally and of which, the UNESCO World Heritage Site Monte San Giorgio, which extends from Switzerland into Italy, is the most important one. Following the discovery of the occurrence of articulated skeletons of marine reptiles in the local mines, large excavations were organized by Bernhard Peyer from the University of Zurich starting 1924. With this collection of articles, we commemorate the successful excavations and research, which initiated the publication of a series of monographies, mostly on the vertebrates but also on the invertebrates of this locality. Especially with the discovery of several remarkably similar Konservat-Lagerstätten in China, the discoveries from Monte San Giorgio gained global relevance. New methodologies such as computed tomography produced a wealth of new data, particularly on endocranial anatomy of several tetrapods.

## Introduction

The Triassic period has received a lot of attention recently because many researchers became interested in key aspects of the rediversification after the Permian–Triassic Mass Extinction (PTME; e.g., Benton et al., 2004; Payne & Clapham, 2012; Scheyer et al., 2014a; Hautmann et al., 2015; but see Nowak et al., 2019 for land plants). Following a phase with widespread anoxia, a disaster fauna established where molluscs such as the bivalve *Claraia* and some ammonoids spread quickly (Airaghi, 1911; Brayard et al., 2006, 2009, 2017; Villier & Korn, 2004). Many Early Triassic faunas accordingly displayed a great dominance (e.g., Friesenbichler et al., 2021). Maybe these peculiar new ecosystems created the conditions enabling the evolution of diverse marine reptiles as well as new groups of osteichthyan fishes, often with dietary specialisations (e.g., Kelley & Pyenson, 2015; Klug et al., 2024a; Scheyer et al., 2014a).

**Fig. 1:**
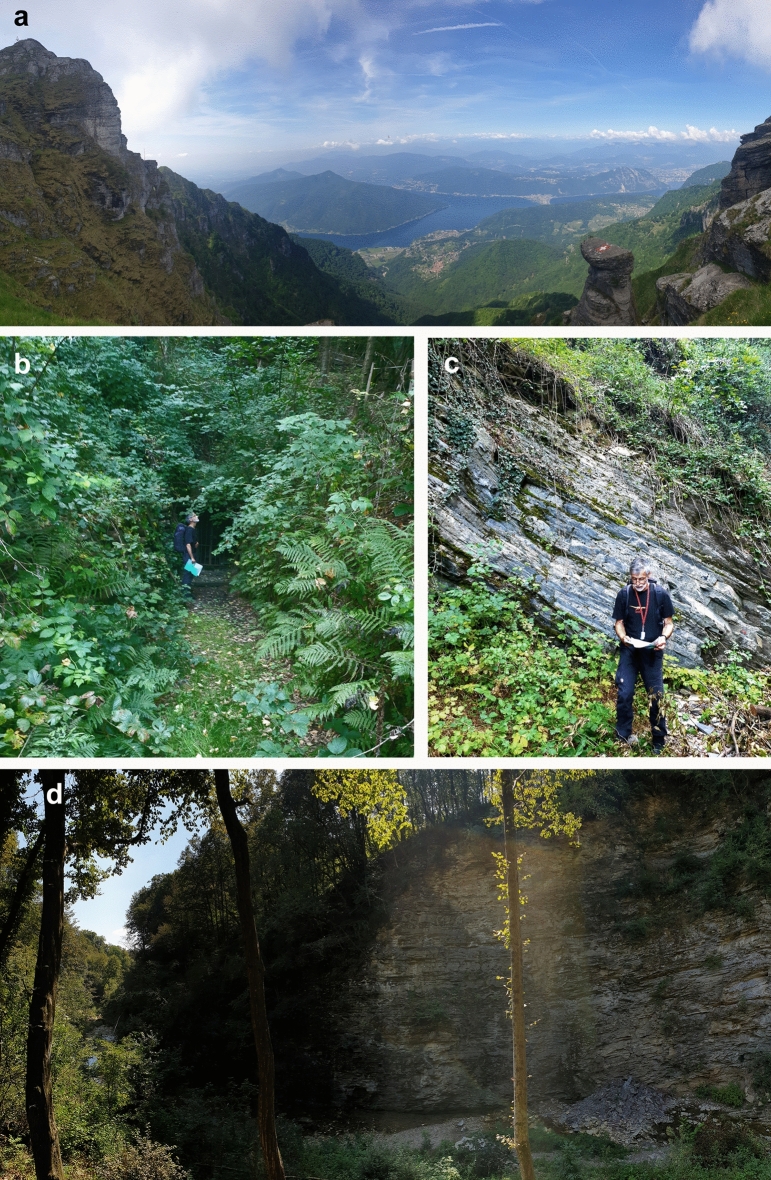
The Swiss side of the UNESCO World Heritage Site of Monte San Giorgio. **a** Monte San Giorgio (surrounded by the Lago di Lugano on its right) seen from the Monte Generoso ridge (summit on the left). **b** Lowermost mine entry at Cava Tre Fontane. **c** Heinz Furrer, the former curator of the Palaeontological Museum of the University of Zurich, guiding a field trip of the Swiss Palaeontological Association in 2020, at the site Acqua del Ghiffo. **d** The natural outcrop of the Kalkschieferzone (uppermost Meride Limestone) at Val Mara, where a rich fish and insect fauna was discovered

During the Middle Triassic, the recovery of marine ecosystems had advanced and quite diverse marine reptiles and fishes populated the epicontinental seas globally (e.g. Klug et al., 2024b). For this context, the mines and excavations of Monte San Giorgio (southern Switzerland) and the adjoining Monte Pravello—Monte Orsa (northern Italy), began to deliver key fossils as early as about 150 years ago, but at first on the Italian territory with the first series of publications on Triassic fossils appearing in the mid and second half of the nineteenth century (Bassani, 1886; Cornalia, 1854; Curioni, 1847, 1863; Stoppani & Bellotti, 1857). In 1919, several decades later, the fossil treasures of Monte San Giorgio were recognized by Bernhard Peyer, who became professor in palaeontology at the University of Zurich in 1943 (Furrer, 2003; Sues, 2024). In 1924, he began with the first excavations at the mine Cava Tre Fontane near Serpiano on the Swiss side (Fig. 1b). This volume is dedicated to the commemoration of the centennial of the beginning of Peyer’s excavations and the according research (e.g., Peyer, 1927, 1930, 1931a, 1931b, 1931c, 1931d, 1931e, 1932, 1934, 1935, 1936a, 1936b; for a more exhaustive and constantly updated list see Albisetti & Furrer, 2023). He later continued very successfully in the same beds of the *Grenzbitumenzone* (today Besano Formation) at the mine Val Porina (Lanz & Felber, 2020), and started the first systematic excavations in fossiliferous beds of the overlying Meride Limestone (Furrer, 1995, 2003, 2024; Kuhn-Schnyder, 1974; Peyer, 1944). His academic offspring and successor Emil Kuhn-Schnyder continued both the excavations and the research, mainly in the *Grenzbitumenzone* (Kuhn-Schnyder, 1974). The great international recognition led the University of Zurich to the foundation of the Paläontologisches Institut und Museum in 1956. Kuhn-Schnyder continued excavations at Cassina (Lower Meride Limestone) until his retirement in 1976 and shortly before published his overview over the research at Monte San Giorgio (Kuhn-Schnyder, 1974). His successor Hans Rieber focused on invertebrates, particularly molluscs (Rieber, 1965, 1968, 1969, 1973a, 1973b). After his retirement, researchers from Zurich, Milano and Lugano contributed significantly to the modernisation of research on Monte San Giorgio fossils, particularly vertebrates, employing state-of-the-art methods such as computed tomography, laminography etc. (e.g., Argyriou et al., 2016; Bastiaans et al., 2023a, 2023b; Beardmore & Furrer, 2015, 2016a, 2016b, 2017, 2019; Beardmore et al., 2012; Bindellini et al., 2021; Ferrante & Cavin, 2023; Ferrante et al., 2023; Hugi, 2011; Hugi & Scheyer, 2012; Hugi et al., 2011; Klein et al., 2023; Kolb et al., 2011; Lautenschlager & Desojo, 2011; Lombardo, 2013; Lombardo & Tintori, 2004; Lombardo et al., 2012; López-Arbarello et al., 2014, 2016, 2019; Maxwell et al., 2013, 2015, 2018; Miedema et al., 2023a, 2023b; Mutter, 2004; Mutter & Herzog, 2004; Neenan et al., 2014; Nosotti, 2007; Nosotti & Rieppel, 2003; Renesto, 2005; Renesto & Avanzini, 2002; Renesto & Stockar, 2018; Renesto et al., 2020; Romano & Brinkmann, 2009; Romano et al., 2016; Scheyer, 2010; Scheyer & Desojo, 2011; Scheyer et al., 2014a, 2014b, 2017; Spiekman & Mujal, 2023; Spiekman et al., 2020a, 2020b, 2021; Stockar, 2010; Stockar & Garassino, 2013; Stockar & Kustatscher, 2010; Stockar & Renesto, 2011; Stockar et al., 2012a, 2012b, 2013; and the papers in this article collection, which are shortly discussed in the next chapter).

**Fig. 2:**
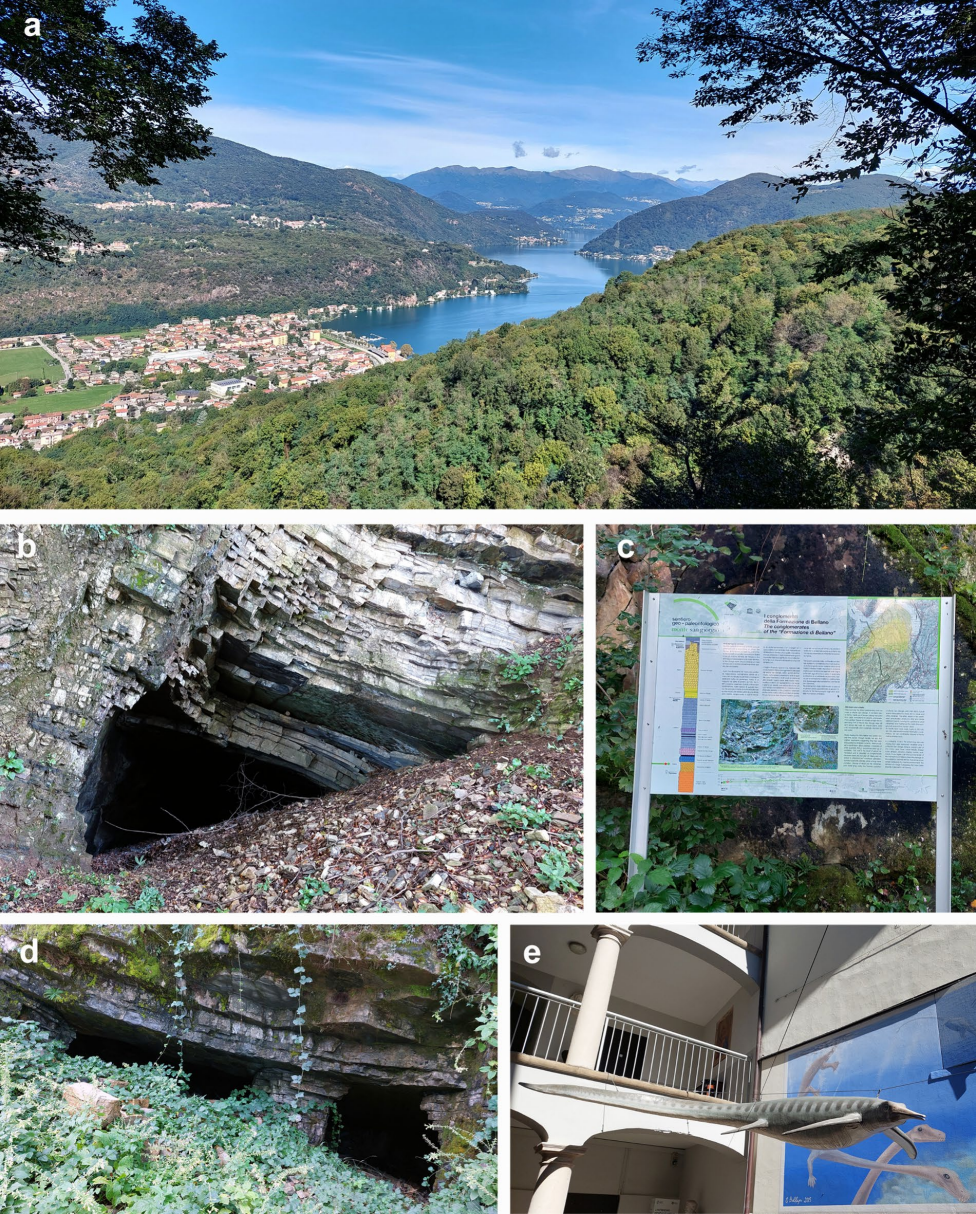
The Italian side of the UNESCO World Heritage Site of Monte San Giorgio. **a** View on Porto Ceresio and its lake), after which *Ceresiosaurus* was named. **b** Entrance of the mine Selvabella Piodelle 3. **c** Plate explaining the conglomerates of the Triassic Bellano Formation. **d** Entrance of the mine Selvabella Piodelle 2. **e** Yard of the Museo Civico dei Fossili di Besano with a model of the ichthyosaur *Besanosaurus* and palaeoart depicting *Tanystropheus* on the wall

21 respectively 14 years ago, the Monte San Giorgio region, first the Swiss and later the Italian side (Figs. 1, 2), were recognized as UNESCO World Heritage Site (Felber et al., 2004; https://whc.unesco.org/uploads/nominations/1090.pdf; see also Furrer, 2003; Felber, 2006; Rieppel, 2019). It is not surprising that the following two decades saw this surge of publications listed in the preceding paragraph. In 2012, the Museo dei fossili del Monte San Giorgio at Meride was opened (see chapter below), which features numerous fossils, illustrations and models. Each year, new state-of-the-art digital installations are being added.

With this editorial chapter, we present this article collection on recent research on Monte San Giorgio to remember the beginning of the excavations on the Swiss side of the mountain 100 years ago. We also want to provide a short overview of the history of palaeontological research on Monte San Giorgio with an emphasis on the Swiss aspects without neglecting the important Italian contributions and fruitful collaborations.

## New palaeontological research on Monte San Giorgio type Lagerstätten

About 170 years after the discovery of the first articulated skeletons in the Monte San Giorgio region and 100 years after the start of the excavations organized by the Palaeontological Institute of the University of Zurich, the digital revolution in combination with new excavations fosters new research on the palaeontology of the region from both the Italian and the Swiss side. In this article collection, we present a series of articles, which cover a broad range of palaeontological topics, reflecting the prolificness and richness of this conservation deposit.

In the article “The marine conservation deposits of Monte San Giorgio (Switzerland, Italy)—the prototype of Triassic black shale Lagerstätten” (Klug et al., 2024b), the pioneering role of Monte San Giorgio and its fossiliferous units is highlighted. The term “Monte San Giorgio type Lagerstätten” is introduced according to the widely used term Burgess type. For many black shales, “Holzmaden type” is proposed and for platy limestones “Solnhofen type”.

In view of the approximately 150 years of research on the Italian side of Monte San Giorgio and 100 years of research on the Swiss side, three articles adequately provided historical overviews of research and excavations (Furrer, 2024), research on fish (Bürgin, 2024) and Bernhard Peyer’s pioneering research (Sues, 2024).

While many Lagerstätten of the Monte San Giorgio type are dominated by vertebrates, an increasing number of invertebrates are discovered, which is reflected in six contributions in this article collection. Two of which describe cephalopods that can be moderately abundant especially in the more carbonatic strata. Despite their dolomitization, they display key characters. Accordingly, Pieroni (2022) described Triassic nautilids and Pohle and Klug (2024) revised the remains of orthoconic cephalopods. Coleoids from other Triassic Lagerstätten were portrayed by Lukeneder et al. (2024) from Austria and Košt’ák et al. (2024) from Slovakia. Montagna et al. (2024) describe a part of a rich, newly excavated insect fauna; insects were poorly documented from Monte San Giorgio before (Bechly & Stockar, 2011; Krzeminski & Lombardo, 2001; Montagna et al., 2017, 2018, 2019; Strada, 2015; Strada et al., 2014). Echinoderms are exceedingly rare and since Jeannet (1933), no further remains have been described. Pieroni (2023) described Cyclida from the Triassic of Italy, although not from Monte San Giorgio.

Fishes played an important role in the Middle Triassic ecosystems of Monte San Giorgio but have been in the shadow of the reptiles for a long time (Bürgin, 2024). The chondrichthyans were described by Kuhn (1946a), Rieppel (1981, 1982) and Mutter (1998). The numerous osteichthyans were studied in detail after the first publications by Brough (1939) and Schwarz (1970): e.g. Rieppel (1985b, 1992); Bürgin et al. (1989); Bürgin (1990a, 1990b, 1992, 1995, 1999a, 1999b, 2024); Lombardo and Tintori (2004); Tintori and Lombardo (2007); Romano and Brinkmann (2009); Lombardo et al. (2012); Lombardo (2013); Maxwell et al., (2013, 2015, 2018); López-Arbarello et al., (2016, 2019); Argyriou et al. (2016); Romano et al. (2016); Renesto et al. (2021a). Arratia et al. (2024) described a new species of the teleostomorph *Marcopoloichthys*.

Recently, the large diversity of sarcopterygians in the Middle Triassic of central Europe was recognized. Following the first publications on sarcopterygians from the Besano Formation (Rieppel, 1980, 1985a) and the description of new coelacanth material from the Meride Limestone (Renesto & Stockar, 2018; Renesto et al., 2021b), Ferrante et al. (2023) revised the moderately common coelacanth *Ticinepomis* and described a new genus and species (*Rieppelia heinzfurreri* Ferrante & Cavin, 2023).

Marine reptiles likely received the greatest attention among the fossils from Monte San Giorgio, mainly because of the abundant ichthyosaurs (e.g., Besmer, 1947; Sander, 1989a; Brinkmann, 1996, 1997, 1998a, 1998b, 1999, 2004; Dal Sasso & Pinna, 1996; Maisch & Matzke, 1997, 1998; Kolb et al., 2011; Pardo-Pérez et al., 2020; Renesto et al., 2020; Bindellini et al., 2021, 2024; Miedema et al., 2023a, 2023b; Klug et al., 2024a) and eosauropterygians (Carroll & Gaskill, 1985; Cornalia, 1854; Hänni, 2004; Hugi, 2011; Hugi & Scheyer, 2012; Hugi et al., 2011; Kuhn-Schnyder, 1962, 1966, 1967, 1987; Nosotti & Rieppel, 2003; Renesto, 1993; Rieppel, 1989, 1994; Sander, 1988, 1989b). In this field, the pioneer Bernhard Peyer contributed a lot to the knowledge of Triassic reptiles (e.g., Peyer, 1927, 1930, 1931a, 1931b, 1931c, 1931d, 1931e, 1932, 1934, 1935, 1936a, 1936b). Even after over a century of research, new aspects about reptiles from Monte San Giorgio are being discovered. For example, Miedema et al. (2023a) examined ontogenetic change in the skull of *Mixosaurus,* whereas Bindellini et al. (2024) provided an in-depth description of the postcranial anatomy of the up to eight meter long *Besanosaurus leptorhynchus*.

Additional reptile groups occur at Monte San Giorgio but much more rarely and yet highly interesting. Particularly noteworthy are the placodonts, thalattosaurs and the iconic long-necked tanystropheids. Thalattosaurs had a global distribution and yet still are understudied (Bastiaans et al., 2023b; Kuhn, 1946b; Kuhn-Schnyder, 1988; Müller, 2005; Nopcsa, 1925; Peyer, 1936a, 1936b; Rieppel, 1987; Rieppel et al., 2005). Klein et al. (2023) studied the bone histology of thalattosaurs for the first time, including sections of *Askeptosaurus italicus* and Bastiaans (2024) provides an overview over thalattosaurs through space and time.

Placodonts were also studied repeatedly (Kuhn, 1942; Kuhn-Schnyder, 1960; Neenan et al., 2014; Peyer, 1931c, 1931e; Pinna, 1992; Scheyer, 2010). Recently, Gere et al. (2024) discovered new aspects in the dietary shift in placodonts.

To put the reptiles from Monte San Giorgio into a broader context, we also included the redescription of *Trachelosaurus* by Spiekman et al. (2024), because it is of relevance for the comparison with other long-necked forms such as *Tanystropheus* (Peyer, 1931b; Wild, 1973, 1980). Gu et al. (2024) provided new information on the dentition of the early branching Chinese ichthyosaur *Chaohusaurus* *zhangjiawanensis*, while López-Arbarello and Brocke (2024) discussed and revised a small ray-finned fish from the Perledo-Varenna Formation, Perledo, Italy and compared it with members of the fish fauna from the Besano Formation. Finally, Klein et al. (2022) introduced the new pachypleurosaur *Prosantosaurus scheffoldi* from Eastern Switzerland and Hu et al. (2024) described a new pachypleurosaur from southwestern China; both studies thus provide comparative material to the Monte San Giorgio pachypleurosaurs.

## The Museum of Fossils from Monte San Giorgio in Meride

The first local Museum of Fossils from Monte San Giorgio was installed by the municipality of Meride with the help of Emil Kuhn-Schnyder and the Palaeontological Institute and Museum, University of Zurich (now the Natural History Museum of UZH), in the heart of the picturesque village, listed in the Federal Inventory of Heritage Sites (ISOS 4002), in 1973 (Kuhn-Schnyder, 1979).

The international recognition of the classic Middle Triassic vertebrate Fossillagerstätte of Monte San Giorgio (Switzerland), already included in the Federal Inventory of Protected Landscapes, Sites and Natural Monuments (BLN 1804), and the adjacent Monte Pravello—Monte Orsa (Italy) was strengthened by its inscription to the UNESCO WHL (World Heritage List; 2003, extended in 2010; see also Furrer, 2003; Felber et al., 2004). Monte San Giorgio yielded the currently best-known record of marine life in the Middle Triassic period (e.g., Klug et al., 2024b), and records important remains of life on land as well (plants: Sordelli, 1879; Peyer, 1944; Wirz, 1945; Stockar & Kustatscher, 2010; terrestrial animals besides insects: e.g., Krebs, 1963, 1965; Nosotti & Rieppel, 2003; Lautenschlager & Desojo, 2011; Jaquier et al., 2017; Miedema et al., 2020; Magnani et al., 2022; Viaretti et al., 2023). The property has produced diverse and numerous fossils, many of which show exceptional completeness and detailed preservation (e.g., Beardmore & Furrer, 2015, 2016a, 2016b, 2017, 2019; Beardmore et al., 2012). The long history of study of the property (Bürgin, 2024; Furrer, 2024; Sues, 2024) and the disciplined management of the resource have created a well-documented and catalogued body of specimens of exceptional quality and are the basis for a rich associated geological literature (World Heritage Committee, 2003, 2010).

**Fig. 3:**
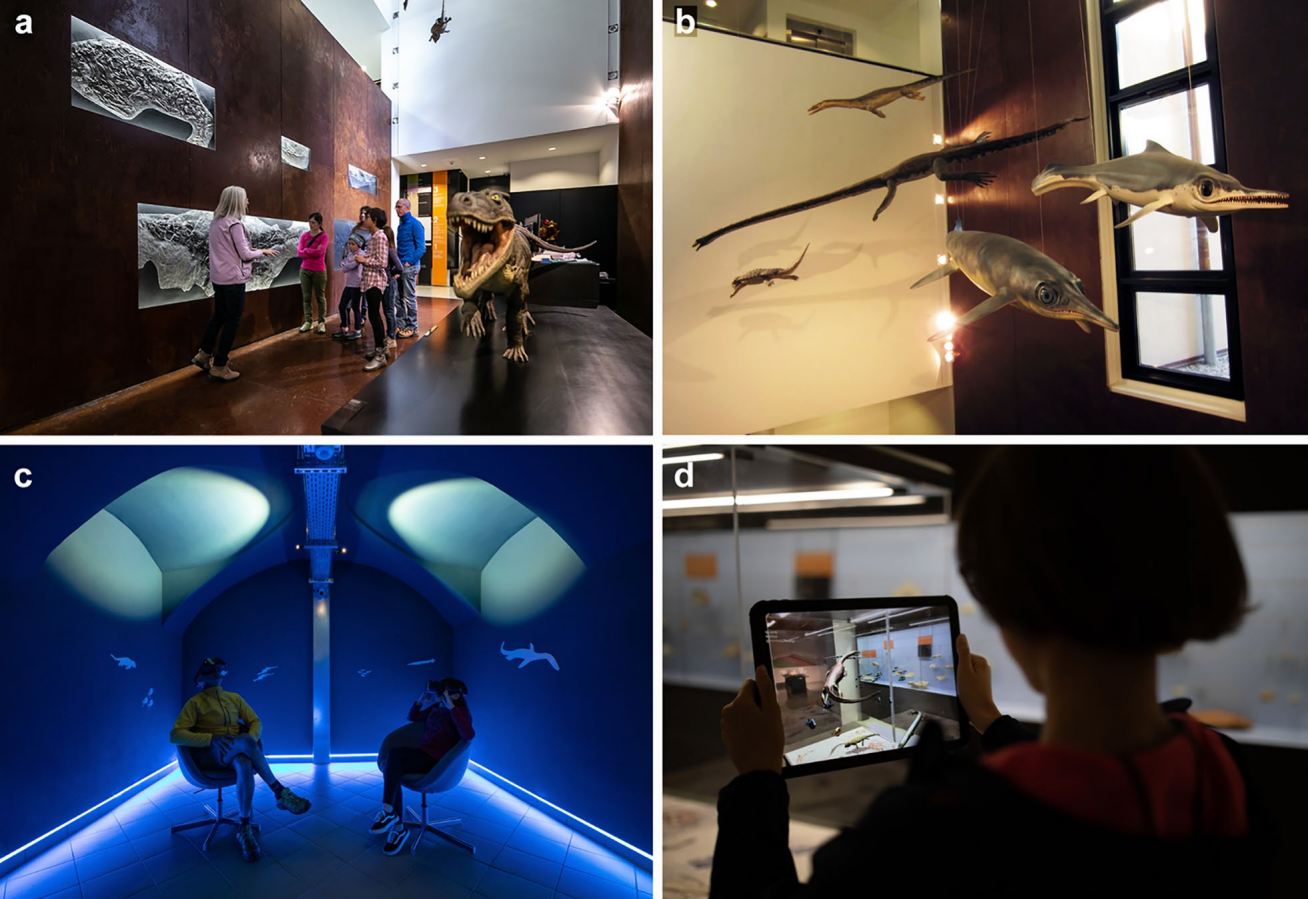
Impressions from the Museo dei fossili del Monte San Giorgio at Meride. **a** Entrance with the model of *Ticinosuchus ferox* by Beat Scheffold. **b** Models of marine reptiles by Beat Scheffold (from left to right: *Cyamodus*, *Tanystropheus*, *Ceresiosaurus*, *Cymbospondylus* and *Mixosaurus*). **c**, **d** Applications of virtual and augmented reality at the museum

The international recognition demanded a new onsite visitor centre and presentation services. In 2012, the new Museo dei fossili del Monte San Giorgio (Fig. 3) opened in a historical lombard-style courtyard house, redesigned by the star architect Mario Botta (Furrer & Vandelli, 2014). The exhibition, initially curated by Heinz Furrer (PIMUZ) and Alberto Bianda (theredbox, Lugano), presents a large variety of fossils spread on four floors, following the stratigraphy from the Middle Triassic to Early Jurassic, mainly originals and a few casts of unique fossils from the PIMUZ, together with attractive life reconstructions and illustrations by Beat Scheffold (Winterthur).

**Fig. 4:**
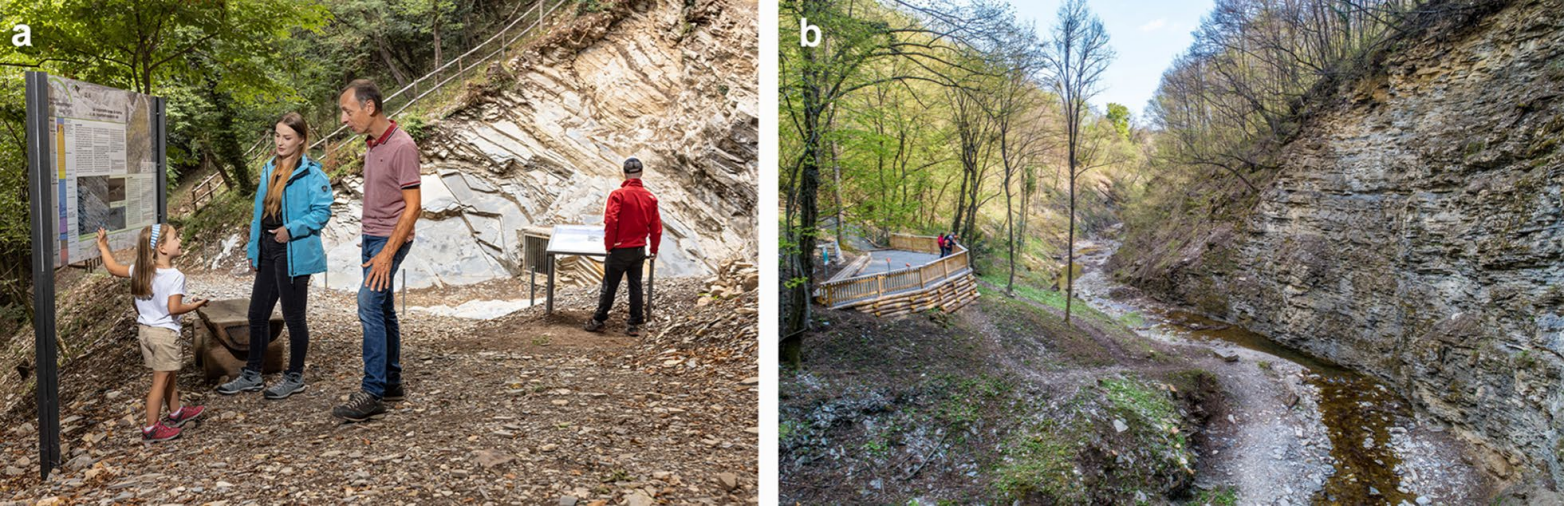
Publicly accessible sites on the Swiss side of Monte San Giorgio. **a** Explanatory plates at Cava superiore beds at Acqua del Ghiffo. **b** Platform at Val Mara, where the exposed Kalkschieferzone (uppermost Meride Limestone) yielded many actinopterygian fish, some crustacean, insect and plant fossils, but only one lariosaurid reptile

The Museo dei fossili del Monte San Giorgio at Meride under the lead of the Fondazione del Monte San Giorgio started with about 10,000 visitors a year and developed successfully to about 20,000 visitors a year, profiting from additional videos, multimedia audio guides for children and adults and innovative three-dimensional animations in augmented, virtual and mixed reality. The actual team led by the site manager Daniele Albisetti and the museum director Luca Zulliger offers various interesting didactic activities in the museum and in the area of Monte San Giorgio:

- Guided tours in the museum, workshops, and field trips (http://www.museodeifossili.ch)
- Educational and experiential paths (http://www.museodeifossili.ch/tracce-fossili.html)
- Geo-palaeontological trail around Monte San Giorgio (http://www.montesangiorgio.org/it/Territorio/Sentiero-geo-paleontologico-transnazionale.html)
- Educational station at the site of the former palaeontological excavations in the lower Meride Limestone (Cava inferiore and Cava superiore beds) at Acqua del Ghiffo (Fig. 4) and Carpanee near Crocefisso
- Panorama platform Val Mara (Fig. 4) at the site of former palaeontological excavations in the upper Meride Limestone (Kalkschieferzone) near Meride
- There is also a project at the entrances of the old oil shale mine at Cava Tre Fontane near Serpiano, for a better presentation of the middle Besano Formation, where from 1907 to 1947 the oil shales (“scisti bituminosi”) and many important fossils were recovered (Furrer, 2016, 2023)

### Further displays of Monte San Giorgio fossils

Monte San Giorgio fossils found their homes in various places, and it is beyond the scope of this editorial to provide a comprehensive list. Here, we file only those museums, which display numerous specimens and taxa. The local museum at Meride plays a key role since it is on sight. Concerning original fossils, however, the Naturhistorisches Museum of the University of Zurich and the Museo di Storia Naturale in Milano display the greatest number of original and important specimens. Both provide a comprehensive overview of the fossil groups of Monte San Giorgio.

*Naturhistorisches Museum der Universität Zürich* The greatest number of holotypes and taxa are likely displayed in the Naturhistorisches Museum der Universität Zürich (by fusion of the Paläontologisches Museum with the Zoologisches, Botanisches, and Anthropologisches Museum). The most remarkable specimens are the complete skeletons of *Tanystropheus, Ticinosuchus, Helveticosaurus*, the thalattosaurs, placodonts, chondrichthyans etc. Some images of the exhibit are provided in Klug et al., (2024b: figs. 1 to 3).

Website: http://www.nmz.uzh.ch.

*Museum of fossils from Monte San Giorgio in Meride/Museo dei fossili del Monte San Giorgio di Meride* This museum and the associated outdoor stations are portrayed in the preceding chapter (Figs. 3, 4).

Websites: http://www.museodeifossili.ch, http://www.montesangiorgio.org/en/.

**Fig. 5:**
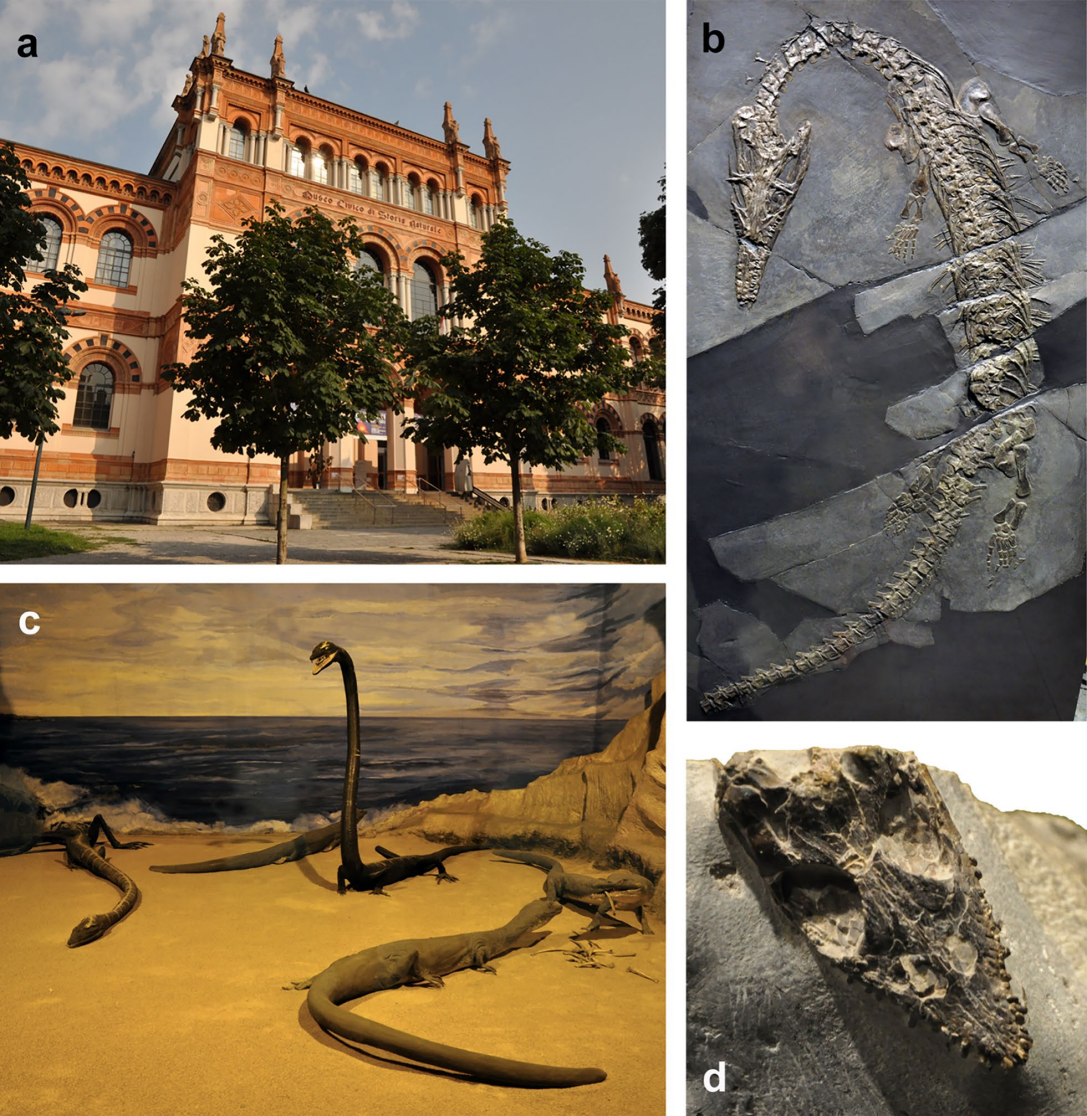
Museo Civico di Storia Naturale di Milano. **a** The beautiful historical façade of the museum. **b** One of the best skeletons of *Askeptosaurus italicus*. **c** The historical diorama featuring *Askeptosaurus* and *Tanystropheus*, showing the earlier notion of a flexible neck and amphibious mode of life of the latter. **d** Holotype of the pachypleurosaurid *Odoiporosaurus terruzzii*

*Museo Civico di Storia Naturale di Milano* This museum exhibits an excellent overview over the fossils of Monte San Giorgio including a huge *Besanosaurus, Askeptosaurus, Tanystropheus* etc. and a nice historic diorama featuring *Tanystropheus* and other marine reptiles on a beach (Fig. 5).

Website: http://www.unescovarese.com/it/14975/Milano-Museo-Civico-di-Storia-Naturale.

**Fig. 6:**
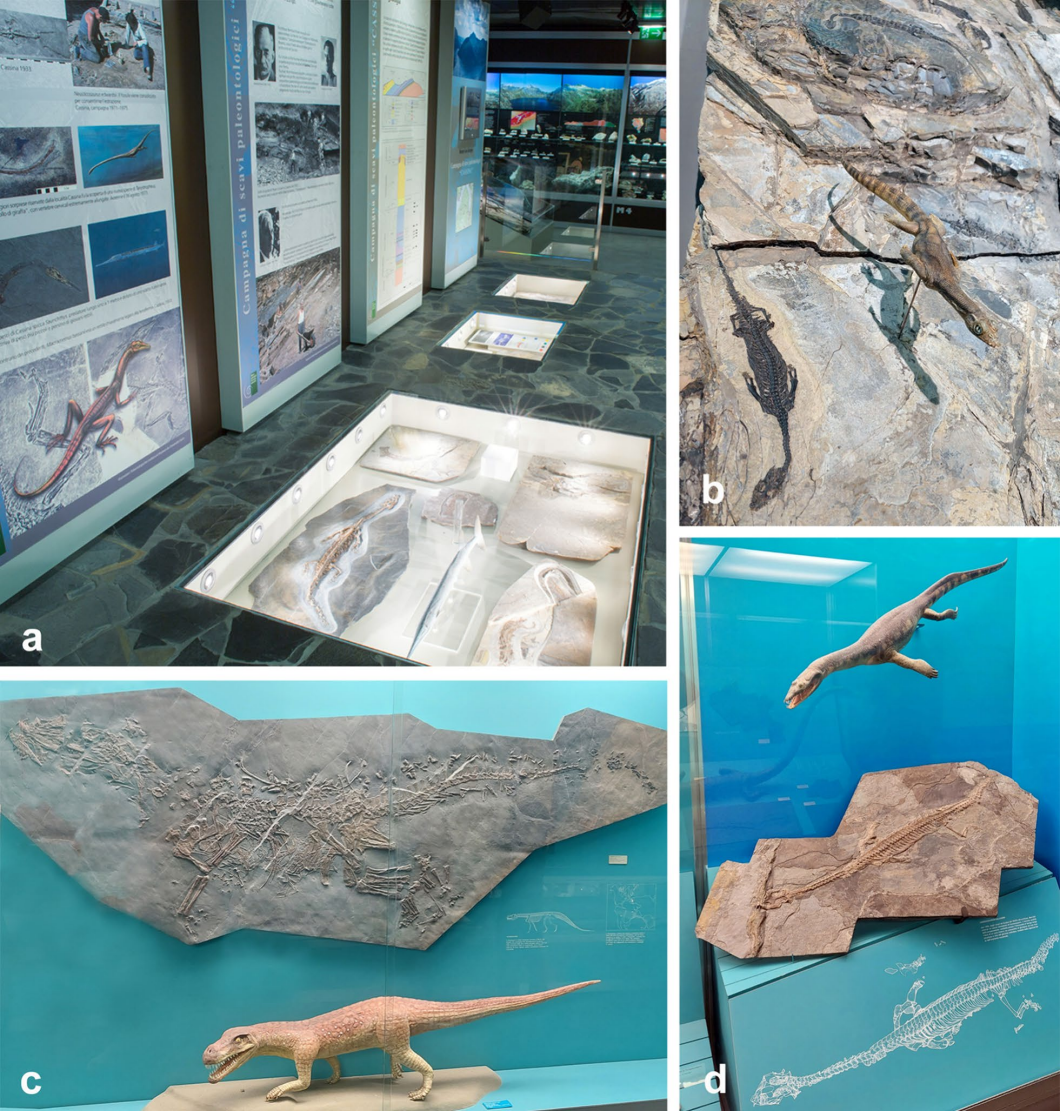
Museo cantonale di storia naturale di Lugano. **a** Displays in the museum. **b** Plate with several skeletons of *Neusticosaurus pusillus*. **c** Cast and model of the rauisuchian *Ticinosuchus ferox*. **d** Skeleton and model (by. B. Scheffold) of *Ceresiosaurus calcagnii*

*Museo cantonale di storia naturale di Lugano* The Cantonal Museum of Natural History researches, documents, and disseminates knowledge about the natural heritage of the Canton of Ticino. The permanent exhibition offers visitors an overview over the fossils from ongoing excavations at Monte San Giorgio (Fig. 6).

Website: http://www.ti.ch/mcsn.

**Fig. 7:**
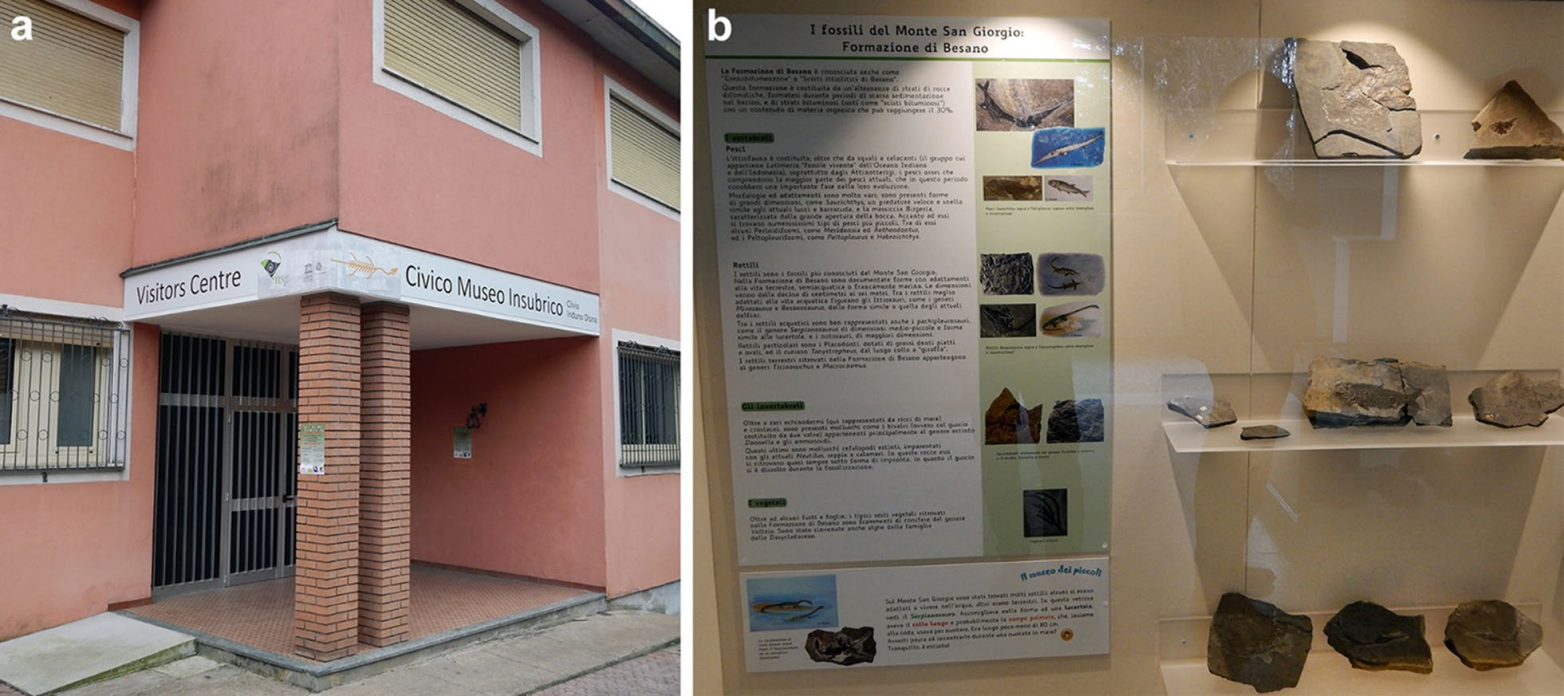
Civico Museo Insubrico di Storia Naturale e Visitor Center Monte San Giorgio UNESCO di Clivio. **a** Entrance. **b** One of the show cases

*Civico Museo Insubrico di Storia Naturale e Visitor Center Monte San Giorgio UNESCO di Clivio* As official visitor centre for the UNESCO world heritage site Monte San Giorgio, this museum will likely increase in importance. The museum houses exhibitions on naturalistic aspects of the Insubric region, with a special focus on the Monte San Giorgio area (Fig. 7).

Website: http://www.unescovarese.com/it/14977/Clivio-VA-Civico-Museo-Insubrico-di-Storia-Naturale-e-Visitor-Center-Monte-San-Giorgio-UNESCO.

*Museo Civico dei Fossili di Besano* Located in a historical building, the museum at Besano has several rooms with displays of Monte San Giorgio fossils and a forum with a big palaeoart s well as a *Besanosauru*s-model (Fig. 2e).

Website: https://museodibesano.it/.

## Conclusions

With this editorial, we introduce an article collection remembering the beginning of scientific excavations by Bernhard Peyer in 1924. This marks an important event in the history of palaeontology of Switzerland because it is still the most important palaeontological site in this country and the only one that received UNESCO world heritage status. Many articles were published in the past decades, highlighting that the combination of the excellent fossil preservation, numerous new methods, and the fame created by the UNESCO-status fostered palaeontological research. We also stress that Monte San Giorgio and the adjoining Monte Pravello—Monte Orsa is a pioneering area, which is the first among numerous other Triassic Lagerstätten worldwide of this unusual combination of facies and fossils, often rich in marine reptiles. This shows that we can expect a wealth of new insights obtained from this area on both sides of the Swiss-Italian border, which hopefully will stimulate research in other regions globally.

## Data Availability

This publication did not use new data.
